# Tumor Budding in Gastric Carcinoma: Beyond Counting Cells at the Invasive Front—A Review of Current Evidence and Biological Perspectives

**DOI:** 10.3390/ijms27093787

**Published:** 2026-04-24

**Authors:** Catalin-Bogdan Satala, Gabriela Gurau, Alina-Mihaela Gurau, Gabriela Patrichi, Daniela Mihalache

**Affiliations:** 1 Faculty of Medicine and Pharmacy, Medical and Pharmaceutical Research Center, “Dunărea de Jos” University of Galati, 800008 Galati, Romania; catalin.satala@ugal.rogabriela.gurau@ugal.ro (G.G.); daniela.mihalache@ugal.ro (D.M.); 2 Department of Pathology, Clinical County Emergency Hospital Braila, 810325 Braila, Romania; 3 The School for Doctoral Studies in Biomedical Sciences, “Dunărea de Jos” University of Galati, 800008 Galati, Romania; 4 The Doctoral School of Medicine and Pharmacy, “George Emil Palade” University of Medicine, Pharmacy, Science and Technology, 540142 Targu Mures, Romania; gabriela.constantin@umfst.ro

**Keywords:** tumor budding, gastric carcinoma, epithelial–mesenchymal transition, tumor plasticity, tumor microenvironment, invasive front, molecular classification

## Abstract

Tumor budding is increasingly recognized as a histopathologic feature associated with invasive behavior in gastrointestinal malignancies. While its prognostic value is well established in colorectal carcinoma, its significance in gastric adenocarcinoma remains less clearly defined because of marked morphologic heterogeneity, variable growth patterns, and the absence of gastric-specific assessment criteria. Multiple studies have associated high budding density with adverse clinicopathologic features, including lymph node metastasis, lymphovascular invasion, advanced tumor stage, and poorer survival, particularly in intestinal-type tumors. However, these associations are more difficult to interpret in diffuse-type and mixed carcinomas, where intrinsic discohesion and architectural variability complicate the distinction between true budding and baseline growth patterns. Beyond prognostic assessment, tumor budding has been linked to localized alterations in cell adhesion, cytoskeletal organization, tumor–stroma interaction, and partial epithelial–mesenchymal transition. Emerging evidence also suggests that its biological significance may differ across molecular subtypes of gastric cancer. This review examines the current evidence on the definition, morphologic spectrum, methodological limitations, and biologic context of tumor budding in gastric adenocarcinoma. We propose that, in gastric cancer, tumor budding is best interpreted not as a uniformly applicable scoring parameter, but as a context-dependent morphologic indicator of invasive tumor remodeling whose meaning varies according to tumor architecture, stromal interface, and molecular subtype.

## 1. Introduction

Tumor budding, defined as the presence of isolated single tumor cells or small clusters of up to four cells at the invasive front, has become an established histopathologic parameter in colorectal carcinoma. Its prognostic relevance, reproducibility, and clinical implications have been consolidated in the International Tumor Budding Consensus Conference (ITBCC), ultimately leading to its integration into reporting recommendations for colorectal cancer [1]. In gastric adenocarcinoma, however, tumor budding occupies a far less defined position. Over the past decade, multiple retrospective cohort studies have reported significant associations between high tumor budding and adverse clinicopathologic features, including lymph node metastasis, lymphovascular invasion, advanced stage, and reduced overall survival [2,3,4]. Despite these observations, tumor budding has not been incorporated into the WHO Classification of Digestive System Tumours (5th edition) nor into CAP reporting protocols for gastric carcinoma. This discrepancy reflects not a lack of evidence, but rather unresolved methodological and conceptual challenges specific to gastric tumor biology [5,6].

Unlike colorectal carcinoma, gastric adenocarcinoma encompasses a broader morphologic spectrum, including intestinal-type, diffuse-type, and mixed growth patterns. In this context, the interpretation of tumor budding is complicated by intrinsic discohesive growth in diffuse-type carcinomas and by the frequent coexistence of heterogeneous architectural patterns within a single tumor [7]. Consequently, the application of colorectal-derived scoring systems to gastric cancer may oversimplify a phenomenon that is biologically and morphologically more nuanced. Recent literature has increasingly linked tumor budding with markers of epithelial–mesenchymal transition (EMT), particularly partial EMT states characterized by reduced membranous E-cadherin expression and redistribution of β-catenin, without complete loss of epithelial identity. This has shifted the discussion from tumor budding as a mere quantitative prognostic parameter toward a potential morphologic correlate of phenotypic plasticity at the invasive front. In gastric cancer, where phenotypic heterogeneity and architectural instability are common, this interpretation may be particularly relevant [8].

Therefore, rather than revisiting tumor budding solely as a prognostic marker, this review examines it as a morphologic phenomenon whose significance in gastric adenocarcinoma depends on the context in which it arises. In this setting, “context-dependent” refers to the fact that the meaning of tumor budding is shaped by histologic subtype, architectural background, tumor–stroma interaction, and molecular subtype, rather than by bud count alone. Thus, similar budding-like structures may reflect localized invasive remodeling in intestinal-type tumors, but may have a different or more limited interpretive value in diffuse-type carcinomas characterized by intrinsic discohesive growth.

To provide a clearer framework, the manuscript is organized in a stepwise manner. We first discuss the definition of tumor budding and the methodological limitations of its assessment in gastric carcinoma. We then examine its morphologic spectrum and spatial heterogeneity, followed by its biologic contextualization, including phenotypic plasticity, tumor–stroma interaction, and molecular classification. Finally, we address translational and technical implications, including digital pathology, therapeutic context, and future directions for gastric-specific standardization.

## 2. Definition and Methodological Challenges in Gastric Carcinoma

Tumor budding in gastric adenocarcinoma is most commonly defined according to criteria extrapolated from colorectal carcinoma, particularly those proposed by the International Tumor Budding Consensus Conference (ITBCC) in 2016. The ITBCC defines a tumor bud as a single tumor cell or a cluster of up to four cells at the invasive front, assessed within a standardized field area of 0.785 mm^2^. While this definition has achieved high reproducibility and clinical validation in colorectal cancer, its direct application to gastric carcinoma remains methodologically unsettled [1].

### 2.1. Architectural Heterogeneity and Definitional Ambiguity

Unlike colorectal adenocarcinoma, gastric cancer frequently displays marked architectural heterogeneity within a single lesion, including gland-forming areas, poorly differentiated clusters, and foci of discohesive growth. This variability complicates the morphological demarcation of a “bud” [9]. In intestinal-type gastric adenocarcinoma, tumor budding typically arises at the periphery of irregular glands. Several studies, including Dao et al., demonstrated that high budding counts correlate with lymph node metastasis and advanced T stage in intestinal-type tumors [2]. However, even in these relatively structured tumors, the distinction between small glandular fragments and true buds can be subtle, particularly in areas of gland fragmentation or stromal desmoplasia [2]. The issue becomes more complex in mixed-pattern carcinomas. Szalai et al. reported that tumor budding retained prognostic significance predominantly in intestinal-type cases, whereas its evaluation in mixed and poorly differentiated tumors showed greater variability. They emphasized that small cell clusters embedded in desmoplastic stroma could represent either budding or intrinsic architectural disintegration [3].

### 2.2. The Diffuse-Type Dilemma

Diffuse-type gastric carcinoma presents a more fundamental challenge. In these tumors, single-cell infiltration is not a peripheral phenomenon but a defining growth pattern. Applying the ITBCC definition in this context risks conflating baseline discohesion with budding activity. Several authors have acknowledged this conceptual limitation [3,10]. They observed weaker prognostic stratification of budding in diffuse-type tumors compared to intestinal-type carcinomas. Yim et al., focusing on early gastric cancer, restricted detailed budding evaluation primarily to non-diffuse tumors due to interpretative difficulty in poorly cohesive carcinoma. This raises an unresolved question: in diffuse-type gastric carcinoma, does tumor budding represent a biologically distinct event, or is it simply an artifact of applying a definition developed for gland-forming tumors? The absence of gastric-specific criteria leaves this distinction largely unresolved [3].

### 2.3. Scoring Variability and Methodological Heterogeneity

Beyond definitional issues, significant heterogeneity exists in scoring methodology across gastric studies. Some cohorts assess budding exclusively on H&E slides [2]. Others incorporate pancytokeratin immunohistochemistry to enhance detection of isolated tumor cells, potentially increasing sensitivity but also altering comparability. Field size selection varies; not all studies adhere strictly to the 0.785 mm^2^ area recommended by ITBCC. Cut-off values for high versus low budding are inconsistent, ranging from arbitrary tertiles to adapted ITBCC thresholds [2,4,11]. Yim et al. proposed a modified tumor budding (mTB) scoring system in early gastric cancer and demonstrated that high mTB independently predicted lymph node metastasis. However, even in that study, the scoring strategy represented an adaptation rather than validation of a gastric-specific consensus framework [12]. A systematic review and meta-analysis of upper gastrointestinal carcinomas highlighted significant inter-study heterogeneity in budding assessment methodology as a major limitation for pooled analysis. Their findings emphasize that while prognostic associations appear consistent, methodological inconsistency reduces interpretative strength [4].

### 2.4. Reproducibility and Interobserver Variability

One of the major strengths of tumor budding in colorectal carcinoma is demonstrated interobserver reproducibility when standardized criteria are applied. In gastric cancer, comparable reproducibility data are limited. Few gastric studies have formally assessed interobserver agreement using kappa statistics. Where evaluated, agreement has been moderate rather than high, particularly in poorly differentiated tumors [9,13,14]. The lack of large-scale reproducibility studies in gastric cohorts contrasts sharply with the colorectal literature and likely contributes to reluctance in guideline adoption. Moreover, reproducibility may be further compromised by: variable inflammatory background obscuring small clusters, desmoplastic stroma mimicking isolated buds, and tissue fragmentation in endoscopic resection specimens. These factors introduce practical diagnostic variability that remains insufficiently quantified in gastric carcinoma [14].

Taken together, these methodological and reproducibility issues help explain why tumor budding has not been incorporated into the WHO Classification of Digestive System Tumours (5th edition) or CAP reporting protocols for gastric carcinoma. More importantly, they suggest that in gastric adenocarcinoma, tumor budding cannot yet be interpreted as a uniformly transferable scoring parameter. Its assessment and significance appear to depend on architectural context, histologic subtype, and the structural conditions of the invasive front. This makes biologic and morphologic contextualization essential for any future gastric-specific framework [5,6].

## 3. Morphologic Spectrum of Tumor Budding in Gastric Carcinoma

Reducing tumor budding to a numerical count obscures its morphologic complexity. In gastric adenocarcinoma, budding is best understood not as a uniform histologic event, but as a spatially and architecturally contextual phenomenon. Its distribution, relationship to glandular structures, association with stromal reaction, and variation across histologic patterns all influence its potential biologic meaning. For this reason, morphologic assessment is not only descriptive, but also essential for understanding why the significance of tumor budding in gastric cancer cannot be assumed to be constant across tumor settings [10,15,16,17].

### 3.1. Budding as a Peripheral Architectural Transition

In intestinal-type gastric adenocarcinoma, tumor budding frequently appears at the interface between distorted glands and desmoplastic stroma. Rather than representing abrupt cell detachment, budding often emerges from glands that gradually lose structural coherence [18]. In a cohort of 128 gastric adenocarcinomas, it was reported that high tumor budding was significantly associated with higher T stage, nodal metastasis, and lymphovascular invasion. Importantly, the authors observed that budding areas often originated from fragmented glandular edges rather than isolated tumor cell islands unrelated to glandular architecture. This supports the interpretation of budding as a progressive architectural transition rather than spontaneous dissociation [2]. Similarly, another study analyzing 292 cases described budding as frequently arising at sites where glandular polarity appeared attenuated and stromal interaction was pronounced. The glands in these regions were not fully lost; rather, they were structurally weakened, producing small protrusions or detached clusters at their periphery. This morphologic sequence—gland attenuation → fragmentation → micro-cluster formation—suggests that budding may represent an intermediate structural state. The tumor core can remain gland-forming, while the invasive margin displays micro-dissociation [3]. This spatial dichotomy challenges the assumption that aggressive features necessarily reflect global dedifferentiation. An unresolved issue is whether budding marks irreversible structural breakdown or a dynamic and potentially reversible transition. Morphology alone cannot answer this, but the coexistence of preserved glandular architecture adjacent to budding supports the idea of localized instability rather than uniform transformation [15].

### 3.2. Spatial Heterogeneity and Sampling Implications

Tumor budding in gastric carcinoma is rarely uniformly distributed. Most studies emphasize the invasive front as the site of assessment, yet even within this region, budding density can vary considerably [1,19]. Szalai et al. observed marked heterogeneity in budding distribution, with high-density budding often confined to focal sectors. They reported that the highest budding counts were frequently identified in limited fields rather than across the entire margin. This focality has direct implications for sampling, particularly in biopsy material and endoscopic resections [3]. Yim et al., studying early gastric cancer treated by endoscopic submucosal dissection, demonstrated that modified tumor budding (mTB) was independently associated with lymph node metastasis. However, they also noted that small specimen size may underestimate budding density, particularly when invasive margins are incompletely represented [12]. This raises a methodological dilemma: if budding is spatially clustered, how reliable is a single field? Unlike colorectal carcinoma, where invasive fronts may be more continuous and circumferential, gastric tumors frequently display discontinuous invasive patterns and mixed architecture. Spatial heterogeneity also raises biological questions [3,20,21,22]. Why do buds cluster in certain regions? Dao et al. reported that budding correlated with increased stromal reaction, suggesting that microenvironmental factors, such as fibroblast activation and extracellular matrix remodeling, may locally promote cellular dissociation [2]. However, few gastric studies have systematically correlated budding density with stromal composition. Thus, budding in gastric cancer should not be interpreted merely as a global tumor attribute but as a spatially constrained phenomenon potentially shaped by local microenvironmental conditions [17,23].

### 3.3. Budding Versus Poorly Differentiated Clusters

The distinction between tumor budding and poorly differentiated clusters (PDCs) deserves closer scrutiny in gastric carcinoma. While both features reflect architectural disorganization, they differ in scale and, possibly, biological implication [3,20,24]. A study that compared the prognostic impact of tumor budding and PDCs in gastric adenocarcinoma demonstrated that high tumor budding was an independent predictor of overall survival in intestinal-type tumors, whereas PDCs showed weaker and less consistent prognostic significance. This finding suggests that micro-dissociation (1–4 cells) may capture a biologically distinct process compared to larger clusters (≥5 cells). The former may reflect early detachment and invasion, while the latter may represent more advanced architectural collapse [3]. In gastric cancer, this distinction is particularly relevant because poorly differentiated areas are common, and over-reliance on cluster size without contextual evaluation may blur meaningful differences. Moreover, in mixed tumors, small clusters may represent transitional states rather than fully undifferentiated growth. The biological implication is subtle but important: budding may reflect micro-invasive competence at the cellular level, whereas larger clusters may reflect broader structural instability. Whether these processes share identical molecular drivers remains unclear in gastric cohorts [3,13,24].

### 3.4. Relationship to Tumor Border Configuration

Although tumor border configuration has not been systematically integrated into gastric cancer staging as in colorectal carcinoma, several studies suggest a relationship between infiltrative growth patterns and budding density [10,25]. Tumors with infiltrative margins exhibited significantly higher budding counts compared to those with pushing borders [2]. This association implies that budding may be a morphologic expression of active stromal invasion rather than passive fragmentation. In gastric cancer, border configuration itself is less standardized as a reporting parameter. However, if budding correlates with infiltrative growth, it may serve as a quantifiable surrogate for invasion pattern. This possibility remains underexplored and represents a gap in the literature [11,26]. Notably, the association between budding and lymphovascular invasion reported in multiple studies further supports the interpretation of budding as part of an invasive phenotype rather than merely an architectural artifact [2,3].

Diffuse-type gastric carcinoma challenges the conceptual framework of tumor budding assessment. Because discohesive growth is intrinsic to this subtype, identifying discrete budding foci becomes problematic. As discussed earlier (Section 2.2), applying cluster-based definitions in this context risks conflating baseline growth pattern with localized invasive activity. Consistent with this limitation, several studies have reported weaker prognostic stratification of tumor budding in diffuse-type tumors compared to intestinal-type carcinomas. In contrast, mixed-type tumors may offer a more informative context, where budding-like structures appear at transitional interfaces between cohesive and discohesive components, potentially reflecting localized architectural instability rather than a uniform invasive mechanism [27,28,29].

These morphologic observations suggest that tumor budding in gastric carcinoma cannot be interpreted as a uniform structural feature. Its appearance depends not only on glandular disintegration or border configuration, but also on the biological setting in which these changes occur. This makes biologic contextualization essential for understanding whether budding reflects active invasive remodeling, localized phenotypic plasticity, or merely structural instability in a given tumor subtype.

## 4. Biological Context of Tumor Budding in Gastric Carcinoma

The morphologic heterogeneity of tumor budding in gastric adenocarcinoma raises a central interpretive question: does budding represent a distinct biologic program of invasion, or is it a context-dependent structural manifestation of different processes occurring at the invasive front? In gastric cancer, this question cannot be addressed by morphology alone. Instead, the biologic significance of tumor budding must be examined in relation to phenotypic plasticity, stromal interaction, and tumor subtype. These considerations are particularly important because most available studies infer biologic meaning from whole-tumor correlations rather than from direct molecular characterization of budding cells themselves. The association between tumor budding and epithelial–mesenchymal transition is frequently assumed in gastrointestinal pathology. However, in gastric adenocarcinoma, this relationship is supported primarily by indirect evidence. Most studies demonstrate correlations at the tumor level, without confirming that budding cells themselves represent a distinct molecular compartment. This distinction is critical, as interpreting tumor budding as a surrogate of phenotypic plasticity requires evidence beyond architectural disintegration at the invasive front [30,31,32,33].

### 4.1. Tumor Budding and Cancer Stem Cell Phenotype: Conceptual Distinctions

The relationship between tumor budding and cancer stem cell (CSC) biology remains an area of conceptual overlap but not equivalence. Cancer stem cells are defined functionally by their capacity for self-renewal and tumor initiation, typically demonstrated through experimental assays rather than purely morphological criteria. In contrast, tumor budding is a histopathologic observation defined by the presence of isolated single cells or small clusters at the invasive front. While several studies have reported enrichment of stemness-associated markers in tumors with high budding activity, this association is generally inferred at the tumor level rather than demonstrated specifically within budding clusters. Morphologically, tumor buds represent a spatially localized pattern of cellular dissociation, whereas CSCs represent a functional subpopulation that may or may not be confined to the invasive front [11,15].

Importantly, tumor budding does not necessarily identify a distinct cellular lineage but may instead reflect a transient phenotypic state influenced by microenvironmental conditions. Thus, although budding and CSC-related features may partially overlap, they should not be considered interchangeable. Tumor budding remains a structural descriptor, whereas CSCs represent a biologically defined functional entity.

### 4.2. Phenotypic Plasticity and the Partial EMT Hypothesis

The concept of partial EMT (pEMT) has provided an appealing framework for interpreting tumor budding. In this model, cells at the invasive front adopt a hybrid phenotype, retaining epithelial markers while acquiring migratory properties. This interpretation is consistent with the view that budding in epithelial malignancies may align with such hybrid states [8]. In gastric adenocarcinoma, however, direct evidence remains sparse. Immunohistochemical studies demonstrate increased expression of EMT-associated transcription factors, such as ZEB1 and Snail, in tumors with high budding scores. Yet these analyses typically quantify expression across whole sections. Few studies have confirmed selective enrichment within budding clusters themselves. Moreover, the assumption that budding equals pEMT risks circular reasoning [11,15]. Budding is defined morphologically as small detached clusters; pEMT is defined biologically as partial loss of epithelial characteristics. The two phenomena are conceptually compatible but not synonymous. Without cell-specific molecular profiling, equating them remains inferential. An additional layer of complexity arises when considering diffuse-type gastric carcinoma. If budding reflects pEMT, then why does diffuse-type carcinoma, characterized by intrinsic discohesion, not consistently demonstrate prognostic stratification based on budding counts [15]? This discrepancy suggests that budding may reflect localized destabilization within otherwise cohesive tumors, rather than a universal EMT state [3]. Reduced membranous E-cadherin expression is frequently reported in tumors with high budding scores [2]. In most reported cases, staining is diminished or discontinuous rather than absent. This nuance is critical. Complete loss of adhesion would imply a transition toward diffuse-type morphology or full EMT, whereas partial reduction suggests transient junctional instability. In intestinal-type gastric carcinoma, where E-cadherin loss is not typically driven by *CDH1* inactivation, attenuation of membranous expression may reflect local environmental modulation rather than genetic loss [34,35]. This supports the idea that budding may correspond to a reversible state of weakened epithelial cohesion rather than irreversible lineage conversion. Thus, adhesion remodeling in budding-rich tumors appears to be graded rather than binary. Whether this represents adaptive invasion or simply mechanical detachment remains unresolved [15,36]. The presence of cytoplasmic or nuclear β-catenin in budding-rich tumors has been interpreted as evidence of pathway activation. A study reported altered β-catenin localization in tumors with high budding density, yet the mechanistic implication of this observation remains ambiguous [3]. In gastric carcinoma, canonical Wnt pathway activation is neither universal nor uniform across subtypes. Nuclear β-catenin accumulation, when observed, may reflect junctional destabilization rather than transcriptional reprogramming. Disruption of adherens junctions can itself liberate β-catenin from the membrane, producing cytoplasmic or nuclear redistribution without necessarily initiating a canonical transcriptional cascade. Therefore, the presence of nuclear β-catenin in budding areas cannot be automatically equated with pathway-driven invasion. The literature has not yet distinguished whether β-catenin redistribution in budding cells is causal, permissive, or epiphenomenal. This uncertainty illustrates a broader issue: molecular correlates of budding are frequently interpreted within established signaling frameworks without spatially resolved validation [11,37,38,39].

A more uncomfortable question must also be considered: could tumor budding in gastric carcinoma simply reflect mechanical fragmentation of unstable glands rather than active phenotypic reprogramming? Desmoplastic stroma, inflammatory infiltration, and glandular distortion can physically separate small cell clusters from larger structures. In such cases, budding might be a structural byproduct rather than a biologically distinct compartment. The fact that high budding correlates with adverse outcomes does not resolve this issue. Correlation with prognosis does not equate to mechanistic significance. It remains possible that budding serves as a morphologic indicator of an already aggressive tumor microenvironment rather than as a driver of invasion.

Bridging this gap requires spatially resolved molecular studies capable of distinguishing whether budding clusters represent unique transcriptional states, selected subclones, or context-dependent structural adaptations at the invasive front [15,40].

## 5. Tumor Budding and the Tumor–Stroma Interface

Because tumor budding is consistently localized to the invasive margin, its interpretation also requires attention to the stromal environment in which it emerges. Beyond tumor-cell intrinsic changes, the gland–stroma interface may provide the structural and signaling conditions that enable detachment, micro-cluster formation, and local invasion. In this sense, the tumor–stroma interface is not merely the background of budding, but a major determinant of its morphologic expression and potential biologic significance.

The invasive front of gastric adenocarcinoma represents a structurally and biologically distinct compartment characterized by active interaction between tumor cells and surrounding stroma. Tumor budding is consistently localized to this interface, suggesting that its emergence cannot be understood independently of stromal context [10,11,41]. Desmoplastic reaction is a frequent feature of budding-rich tumors. In gastric carcinoma, particularly of intestinal type, the invasive margin often demonstrates dense fibroblastic proliferation, extracellular matrix deposition, and variable inflammatory infiltration. These stromal components alter the mechanical and biochemical environment surrounding tumor glands. Small epithelial clusters at the invasive front are frequently embedded within newly formed collagen bundles or in close proximity to activated fibroblasts. Cancer-associated fibroblasts (CAFs) play a central role in shaping the invasive front. CAFs produce extracellular matrix proteins, matrix metalloproteinases, and paracrine factors that influence tumor cell adhesion and motility. Although direct studies correlating CAF density with tumor budding in gastric cancer are limited, broader invasive-front analyses demonstrate that fibroblast-rich stroma is associated with infiltrative growth patterns and adverse outcomes [17,42,43]. It is plausible that budding represents the morphologic manifestation of epithelial cells responding to stromal remodeling rather than initiating invasion autonomously.

In addition to structural remodeling, soluble microenvironmental factors may further contribute to the emergence of tumor budding. Signaling pathways involving transforming growth factor-β (TGF-β), Wnt ligands, epidermal growth factor (EGF), and various cytokines and chemokines have been implicated in modulating cell adhesion, motility, and phenotypic plasticity at the invasive front. Although direct evidence specifically linking these pathways to budding clusters in gastric carcinoma remains limited, their established roles in invasion and epithelial–mesenchymal transition suggest that tumor budding may arise within a permissive signaling niche shaped by these factors.

Matrix composition and stiffness may further influence budding formation. Increased extracellular matrix deposition can impose physical constraints on glandular structures, promoting fragmentation of attenuated epithelial edges. At the same time, altered integrin signaling in response to matrix components may facilitate localized detachment and migration. Whether budding cells exhibit distinct integrin expression profiles compared to central tumor cells has not been systematically investigated in gastric carcinoma, but this represents a potential mechanistic link between stromal architecture and epithelial dissociation. The inflammatory component of the tumor microenvironment introduces additional variability. In MSI-high and EBV-associated gastric carcinomas, dense lymphoid infiltrates may obscure small epithelial clusters, complicating budding assessment [43,44,45,46,47]. Beyond technical considerations, inflammatory cytokines and immune–epithelial interactions may modulate adhesion molecule expression and cellular cohesion at the invasive front. The degree to which immune-rich microenvironments suppress or promote budding remains unclear. Lymphovascular structures at the invasive front provide another relevant interface. Budding-rich tumors frequently demonstrate lymphovascular invasion, and small tumor clusters are often observed adjacent to vascular channels. Whether budding clusters represent precursors to vascular invasion or simply co-localize within permissive stromal niches remains unresolved. Morphologically, the proximity of buds to lymphatic spaces suggests that stromal remodeling may create microenvironments conducive to vascular entry. Importantly, the tumor–stroma interface in gastric carcinoma varies according to histologic subtype. In intestinal-type tumors, desmoplastic reaction is often pronounced, creating a clearly demarcated invasive front [3,10,40]. In diffuse-type carcinoma, stromal interaction is more diffuse and interstitial, without discrete gland–stroma boundaries. This structural difference may explain why budding is more morphologically identifiable and prognostically relevant in gland-forming tumors. Despite consistent localization of budding to stromal interfaces, few gastric studies have quantitatively integrated stromal metrics with budding density. Parameters such as tumor–stroma ratio, fibroblast activation markers, or extracellular matrix signatures have rarely been analyzed alongside budding counts. Without such integrative analysis, the contribution of stromal context to budding formation remains largely speculative. Taken together, tumor budding in gastric adenocarcinoma appears tightly linked to the invasive front microenvironment. Rather than representing a purely epithelial phenomenon, budding likely reflects dynamic interaction between epithelial instability and stromal remodeling. Clarifying this relation requires coordinated morphologic, molecular, and spatial analysis of both compartments [10,23,48].

While stromal interaction helps explain the local emergence of tumor budding at the invasive front, it does not fully account for the marked variability in its morphologic and prognostic significance across gastric cancers. This broader heterogeneity suggests that tumor budding should also be interpreted in relation to molecular subtype.

## 6. Tumor Budding in the Context of Molecular Classification

**Figure 1:**
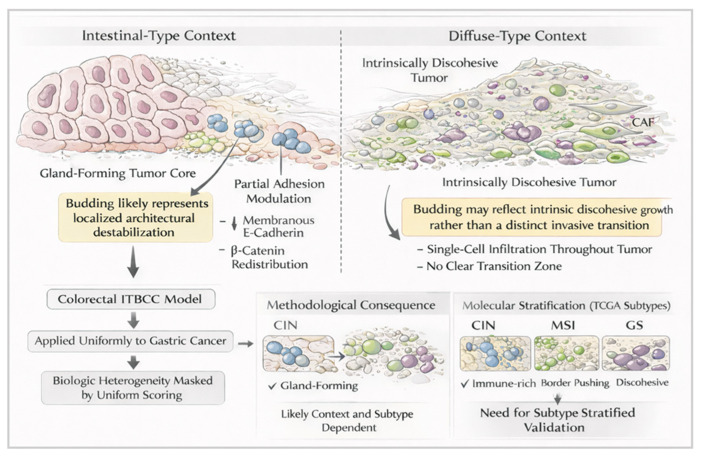
Context-dependent interpretation of tumor budding in gastric adenocarcinoma. Tumor budding does not carry uniform biological meaning across gastric cancer subtypes. In intestinal-type tumors, budding appears at the invasive front as a localized architectural destabilization associated with decreased membranous E-cadherin and β-catenin redistribution within an otherwise cohesive gland-forming tumor. In diffuse-type carcinoma, where discohesion is intrinsic and widespread, identical morphologic criteria may reflect baseline growth pattern rather than a distinct invasive transition. Uniform application of colorectal-derived scoring frameworks may therefore mask biologic heterogeneity, highlighting the need for subtype-stratified validation in gastric adenocarcinoma.

Large-scale molecular profiling studies have fundamentally reshaped the understanding of gastric adenocarcinoma heterogeneity. The TCGA and ACRG classifications have demonstrated that gastric cancer is not a single disease but a composite of biologically distinct entities defined by genomic instability patterns, signaling alterations, and transcriptional programs [49,50]. Despite this stratification, tumor budding has rarely been evaluated within molecularly annotated cohorts, creating a gap between histomorphology and genomic taxonomy. The TCGA framework identifies four principal subtypes: Epstein–Barr virus (EBV)-positive, microsatellite instability (MSI), chromosomal instability (CIN), and genomically stable (GS) tumors. Each subtype exhibits distinct structural and molecular characteristics that may influence the emergence and interpretation of tumor budding [11,49,50]. CIN tumors, which represent the largest subgroup and frequently correspond to intestinal-type histology, are characterized by extensive somatic copy number alterations and amplifications involving receptor tyrosine kinases such as *ERBB2*, *EGFR*, and *MET*. These tumors typically demonstrate gland-forming architecture with variable infiltrative growth patterns. In this structural context, tumor budding may represent focal architectural destabilization superimposed on otherwise cohesive epithelial structures. Chromosomal instability may contribute to intratumoral heterogeneity, potentially generating subclonal populations with altered adhesion or invasive properties at the tumor margin. However, direct correlation between CIN status and budding density has not been systematically assessed in large, genomically annotated cohorts. The GS subtype, enriched for diffuse-type histology and characterized by alterations in *CDH1* and *RHOA*, presents a distinct scenario. *CDH1* inactivation leads to impaired cell–cell adhesion at a fundamental level, producing the discohesive growth pattern typical of diffuse carcinoma. In such tumors, single-cell infiltration is intrinsic to baseline architecture rather than a localized invasive adaptation [11,49,50,51,52]. The conceptual overlap between diffuse growth and budding challenges the applicability of identical scoring frameworks. If budding reflects a transition from cohesive to discohesive growth, its relevance may be limited in tumors where discohesion is already constitutive. The reduced prognostic stratification of budding in diffuse-type tumors reported in several cohorts is consistent with this interpretation. *RHOA* mutations, also enriched in GS tumors, affect cytoskeletal organization and cell motility. Whether *RHOA*-altered tumors exhibit distinct budding patterns has not been specifically evaluated, but cytoskeletal dysregulation may influence invasive morphology independently of classical adhesion molecule loss. MSI-high tumors introduce additional complexity. These tumors are characterized by hypermutation and prominent immune infiltration. The dense inflammatory background may obscure small tumor clusters and complicate budding assessment on routine H&E sections [3,11,18,50,51,52,53,54]. Moreover, MSI tumors often display medullary or pushing growth patterns rather than highly infiltrative borders. It remains unclear whether budding density retains prognostic significance within this molecular context. Stratified analysis of budding in MSI versus microsatellite-stable tumors has not been consistently performed. EBV-positive gastric carcinomas represent a smaller but biologically distinct group, characterized by recurrent *PIK3CA* mutations and *PD-L1/PD-L2* amplification. These tumors frequently exhibit lymphoepithelioma-like morphology with intense immune infiltration. Whether such tumors demonstrate meaningful budding patterns has not been systematically investigated, and inflammatory obscuration may further complicate interpretation [45,50,54,55]. The ACRG classification adds a complementary perspective by incorporating transcriptional features and survival stratification. The ACRG “MSS/EMT” subtype, associated with diffuse histology and poor prognosis, is defined by enrichment of EMT-related gene expression signatures. In this context, the relationship between molecular EMT signatures and morphologic budding requires careful consideration. If a tumor already exhibits a global EMT-like transcriptional profile, localized budding may not provide additional discriminatory value. Conversely, in tumors without strong EMT signatures, focal budding might represent a localized shift toward mesenchymal-associated programs. Importantly, most existing budding studies lack integrated genomic annotation. As a result, potential associations between budding density and specific molecular alterations, such as *TP53* mutation status, *ERBB2* amplification, or *RHOA* mutation, remain unexplored. Without molecularly stratified analyses, it is difficult to determine whether budding is enriched in particular genomic contexts or whether it represents a cross-subtype morphologic expression of invasive architecture [11,39,49,51,56,57,58,59]. The integration of tumor budding assessment with molecular classification frameworks would allow several clarifications. First, it could establish whether budding frequency differs significantly across TCGA subtypes. Second, it could determine whether budding provides incremental prognostic value within specific molecular categories. Third, it could clarify whether budding corresponds to defined transcriptional states or merely reflects architectural manifestation of diverse underlying genomic alterations. Until such integrative studies are performed, tumor budding remains largely detached from molecular stratification paradigms. Bridging histomorphology with genomic context represents an essential next step in determining whether budding is a subtype-specific phenomenon or a broadly applicable indicator of invasive remodeling across molecular categories (Figure 1) [11,49,50,60,61,62,63].

## 7. Digital Pathology and Quantification Challenges

The methodological and biologic heterogeneity of tumor budding in gastric adenocarcinoma has direct implications for its quantification. Because budding in gastric cancer is often spatially uneven, architecturally context-dependent, and variably expressed across histologic and molecular settings, its assessment remains highly sensitive to field selection and observer interpretation. These limitations make digital pathology an attractive tool, but also highlight why simple transfer of colorectal-based digital approaches may be insufficient in gastric tumors. In colorectal carcinoma, the invasive front often forms a relatively continuous circumferential boundary, facilitating systematic identification of budding-rich regions. In gastric adenocarcinoma, particularly in large or mixed-pattern tumors, the invasive interface may be discontinuous, irregular, or multifocal [1,4,9,11,64]. Budding density can vary substantially across different sectors of the same tumor. Consequently, hotspot selection may reflect sampling bias rather than global tumor behavior. Field size variability further complicates interpretation. While the ITBCC framework defines a standardized field area for colorectal cancer, gastric studies have not consistently adhered to identical magnification or area calibration. Even small variations in field diameter may significantly alter bud counts in tumors with focal clustering. Without uniform digital calibration across microscopes or scanners, inter-institutional comparability remains limited. Digital pathology offers potential solutions but introduces new challenges. Whole-slide imaging enables systematic scanning of the entire invasive front, theoretically reducing subjective hotspot bias. Automated detection algorithms can identify small epithelial clusters based on size and morphological criteria [1,4,11,36]. However, gastric adenocarcinoma presents specific obstacles for algorithmic quantification. First, stromal complexity in gastric tumors is highly variable. Dense desmoplasia, mucin pools, inflammatory infiltrates, and artifact from biopsy fragmentation can mimic or obscure small tumor clusters. Algorithms trained on colorectal carcinoma may misclassify mucin-associated epithelial fragments or inflammatory cell aggregates in gastric specimens [65,66,67,68]. Second, diffuse-type carcinoma complicates digital segmentation. Isolated single cells dispersed throughout the stroma may fulfill numerical definitions of buds but do not necessarily represent focal invasive transition. Distinguishing intrinsic discohesive growth from true budding requires architectural context that current automated systems may struggle to interpret. As discussed in Section 5, the use of cytokeratin immunohistochemistry may increase detection sensitivity but also introduces a risk of overestimation. In the context of digital pathology, this limitation may be amplified, as automated systems may detect epithelial fragments or artifacts without sufficient architectural context. Reproducibility studies in digital budding assessment for gastric carcinoma remain limited [65,66,69]. While AI-assisted quantification has shown promise in colorectal cancer, validation datasets in gastric carcinoma are sparse and often small. Given the greater architectural diversity of gastric tumors, algorithm training must account for subtype-specific morphology. The potential advantage of digital assessment lies in comprehensive spatial analysis rather than simple automation of hotspot counting. Whole-slide quantification of budding distribution, spatial clustering, and proximity to lymphovascular structures could provide more nuanced characterization than manual counting within a single field [67,68,70]. However, such approaches require standardized annotation protocols and large, well-annotated training cohorts. At present, digital pathology represents a promising but unvalidated adjunct for tumor budding assessment in gastric adenocarcinoma. Without rigorous validation and subtype-aware algorithm design, automation risks reproducing existing methodological ambiguities at scale [64,65,66,67,68,69,70].

## 8. Therapeutic Relevance and Current Predictive Limitations

If tumor budding in gastric adenocarcinoma reflects biologically relevant invasive remodeling, an important question is whether this morphologic feature also carries therapeutic significance. At present, however, most available studies have focused on prognostic associations rather than on treatment response [3,11,50,71]. As a result, the possible predictive value of tumor budding across molecularly defined therapeutic subgroups remains largely hypothetical. In HER2-positive gastric carcinoma, which is enriched within chromosomal instability (CIN) tumors, amplification of *ERBB2* defines eligibility for trastuzumab-based therapy. Budding has not been systematically analyzed in relation to HER2 status. However, given that CIN tumors frequently display gland-forming architecture with focal infiltrative margins, it is conceivable that budding density may vary within this subgroup. Whether HER2-amplified tumors with high budding exhibit differential response to anti-HER2 therapy has not been addressed in available cohorts [13,43,50,72,73,74]. Microsatellite instability-high (MSI-H) tumors, characterized by high mutational burden and responsiveness to immune checkpoint inhibitors, represent another relevant therapeutic subgroup. MSI-H gastric carcinomas often demonstrate prominent lymphoid infiltration and may exhibit pushing rather than infiltrative borders. The relationship between budding density and immune-rich microenvironments has not been clearly defined. If budding reflects localized invasive remodeling, its frequency in MSI-H tumors could theoretically be reduced; however, stratified analyses are lacking. Furthermore, whether budding correlates with response to PD-1/PD-L1 blockade remains unknown [43,44,75,76]. Diffuse-type and genomically stable tumors, frequently associated with *CDH1* and *RHOA* alterations, generally exhibit limited benefit from HER2-targeted strategies and have distinct patterns of chemotherapeutic response. In this context, budding may overlap biologically with intrinsic discohesive growth rather than representing an independent therapeutic biomarker. Current data do not support the use of budding as a predictive marker in this subgroup. The role of tumor budding in the neoadjuvant setting has also received limited attention. Perioperative chemotherapy induces variable tumor regression patterns in gastric carcinoma. It is unclear whether pre-treatment budding density predicts response, or whether residual budding after therapy reflects treatment-resistant subclones. Few studies have systematically evaluated budding in post-neoadjuvant resection specimens, and standardized scoring in regressed tumors presents additional challenges due to therapy-induced fragmentation and stromal fibrosis. At present, tumor budding should not be interpreted as a predictive biomarker for targeted or immunotherapeutic strategies in gastric adenocarcinoma [11,13,50,77,78]. The absence of prospective, treatment-stratified analyses limits any definitive conclusions. However, its consistent association with invasive phenotype raises the possibility that budding may reflect tumor compartments enriched for therapy-resistant or migratory cell populations. This hypothesis requires direct investigation within molecularly annotated and treatment-annotated cohorts. Future studies integrating budding assessment with therapeutic response data could clarify whether this morphologic parameter has value beyond prognostic correlation. Until such evidence emerges, tumor budding remains primarily a structural and biological descriptor rather than a clinically actionable predictive marker [13,14,79].

## 9. Future Directions: Toward Standardization and Biological Clarification

Future work on tumor budding in gastric adenocarcinoma should move beyond simple adaptation of colorectal-based frameworks. A major priority is the development of gastric-specific assessment criteria that account for architectural heterogeneity, particularly the interpretive differences between intestinal-type, diffuse-type, and mixed tumors. Such efforts should include dedicated reproducibility studies, clearer guidance on field selection and cut-off thresholds, and more precise distinction between true budding and baseline discohesive growth. These issues are especially relevant in early gastric cancer, where budding has been proposed as an additional factor in refining the indication for surgery after endoscopic resection, but where broader validation remains necessary before routine implementation [80].

A second priority is biologic validation. Although tumor budding has been associated with phenotypic plasticity, stromal remodeling, and subtype-specific patterns, most current evidence remains indirect and based on whole-tumor correlations. Spatially resolved approaches, including single-cell and spatial transcriptomic strategies as well as targeted analysis of invasive-front compartments, are needed to determine whether budding reflects a distinct transcriptional state, a microenvironmentally conditioned adaptation, or a structural consequence of glandular instability [81,82,83,84]. In parallel, future studies should further clarify how budding relates to gastric-lineage and differentiation-associated markers, including mucin phenotype and VSIG1-associated pathways, particularly in relation to subtype-specific architectural remodeling [85,86].

Finally, translational relevance will require integrated validation in large, well-annotated cohorts. Future studies should combine histology, molecular subtype, treatment data, and digital whole-slide analysis in order to determine whether tumor budding can be assessed reproducibly and interpreted in a clinically meaningful way across different gastric cancer settings. Prospective, subtype-stratified studies will be essential to establish whether tumor budding provides added value beyond established clinicopathologic parameters and whether it can eventually contribute to risk stratification or management decisions in selected patient groups.

## 10. Conclusions

Tumor budding in gastric adenocarcinoma should not be viewed simply as an imported prognostic metric derived from colorectal cancer. Current evidence indicates that its significance in gastric tumors is shaped by architectural heterogeneity, histologic subtype, stromal interaction, and molecular background. In this setting, budding appears most informative when interpreted as a context-dependent morphologic indicator of invasive tumor remodeling rather than as a uniformly applicable scoring parameter.

Available studies support an association between high tumor budding and adverse clinicopathologic features, particularly in intestinal-type tumors, but substantial limitations remain. These include the absence of gastric-specific assessment criteria, limited reproducibility data, and the unresolved question of whether budding represents active phenotypic plasticity, microenvironmentally conditioned adaptation, or structural fragmentation in different tumor settings.

Taken together, current data support a more nuanced interpretation of tumor budding in gastric carcinoma: not as a single biologic entity with fixed meaning, but as a spatially and biologically contextual feature whose value depends on the setting in which it arises. Further progress will depend on gastric-specific standardization, spatially resolved biologic validation, and integrated clinicopathologic studies capable of defining when tumor budding offers true added value in gastric cancer assessment.

## Data Availability

No new data were created or analyzed in this study. Data sharing is not applicable to this article.
